# Motor Improvements Following Non-Invasive Brain Stimulation with kTMP: A Case Study of Cerebellar Stroke

**DOI:** 10.1007/s12311-026-02078-z

**Published:** 2026-09-11

**Authors:** Amanda Chirino-Pérez, Christina M. Merrick, Fredy Miranda-Casasola, Ludovica Labruna, Daniel Sheltraw, Richard B. Ivry

**Affiliations:** 1 Department of Psychology, University of California, Berkeley, CA 94720 USA; 2 Magnetic Tides Inc, Berkeley, CA 94704 USA; 3 Neuropsychology Laboratory, Physiology Department, School of Medicine, National Autonomous University of Mexico, Mexico City, 04510 Mexico; 4 Department of Neuroscience, University of California, Berkeley, CA 94720 USA

**Keywords:** Cerebellar ataxia, Stroke, Non-invasive brain stimulation, Postural stability, Gait

## Abstract

Cerebellar ataxia results in debilitating impairments in balance, gait, and cognition, with no established effective treatments. Kilohertz transcranial magnetic perturbation (kTMP) is a novel subthreshold non-invasive brain stimulation (NIBS) method that delivers strong, subthreshold electric fields to the cerebellar cortex without discomfort. We present the case of a 78-year-old patient with cerebellar ataxia following hemorrhagic stroke who underwent amplitude-modulated kTMP (AM-kTMP) targeted to vermal and paravermal cerebellar regions. The design involved an open-label phase followed by a fixed-sequence sham-active comparison. Active AM-kTMP, delivered as part of a NIBS-training protocol, produced improvements on clinical scales of ataxia alongside enhanced gait stability, with no device-related adverse reactions, representing a promising NIBS intervention for cerebellar disorders.

**Supplementary Information:**

The online version contains supplementary material available at https://doi.org/10.1007/s12311-026-02078-z.

## Introduction

The treatment of cerebellar ataxia (CA) presents a formidable clinical challenge. At present, only a single drug has received FDA approval for treating ataxia, with available evidence suggesting modest efficacy that may be offset by the potential for serious side effects [1]. Invasive deep brain stimulation (DBS) has been used in the treatment of CA and other movement disorders associated with cerebellar function, with implants placed either in the cerebellum or thalamic nuclei that receive input from the deep cerebellar nuclei [2]. However, DBS entails significant surgical risk and is likely to be difficult to employ at scale.

The lack of established treatments has led to considerable interest in the potential of non-invasive brain stimulation (NIBS) as a treatment for CA [3]. For example, patients with SCA3 showed a ~ 15% improvement on a standard ataxia rating scale following a 2-week protocol involving repetitive TMS [4]. Improvements have also been observed in studies in which tDCS was used as the NIBS intervention [5]. However, there are also reports of no measurable change following NIBS interventions [3].

There are inherent limitations with current NIBS methods when used to target the cerebellum. Electrical induction methods such as tDCS only induce a small, subthreshold E-field in the brain [6]. This challenge is magnified when targeting deeper structures such as the cerebellum, with average E-field values ranging approximately 0.3–0.4 V/m [7]. While TMS can reach suprathreshold levels, the TMS waveforms are brief pulses (possibly repeated) of fixed shape resulting in a broadband and inflexible stimulus. Moreover, TMS can be difficult to tolerate when applied over the cerebellum due to contraction of neck muscles.

With these limitations in mind, we developed a new NIBS method, kilohertz transcranial magnetic perturbation (kTMP) in which a high-current amplifier is used to drive a coil placed on the scalp. Power considerations require that the system operates in the kHz range. By leveraging magnetic induction, kTMP induces significantly stronger subthreshold E-fields than traditional tES; our current kTMP system can reach up to 10 V/m at the cortical surface and 6 V/m at the cerebellar cortex. Amplitude modulation (AM) of the carrier frequency can be tailored to target specific physiological frequencies.

Studies in both human and non-human models have shown that neurons respond to subthreshold kHz stimulation [8]. In studies involving healthy young adults, we have shown that non-modulated kTMP over the primary motor cortex (M1) increases excitability for up to 40 min [9]. In pilot work from our group, AM-kTMP in the beta range attenuated motor skill learning in healthy young adults whereas 3 Hz AM-kTMP combined with movement therapy improved upper limb function in chronic stroke patients. Across 433 sessions involving 143 healthy adults and chronic stroke patients, kTMP directed at various brain targets has proven to be safe, highly tolerable, and perceptually indistinguishable from sham stimulation, with no device-related adverse reactions or abnormal muscle activations recorded [10].

Here, we present the first application of kTMP as an intervention for cerebellar ataxia.

## Case Report

### Patient Presentation and Baseline

**Fig. 1:**
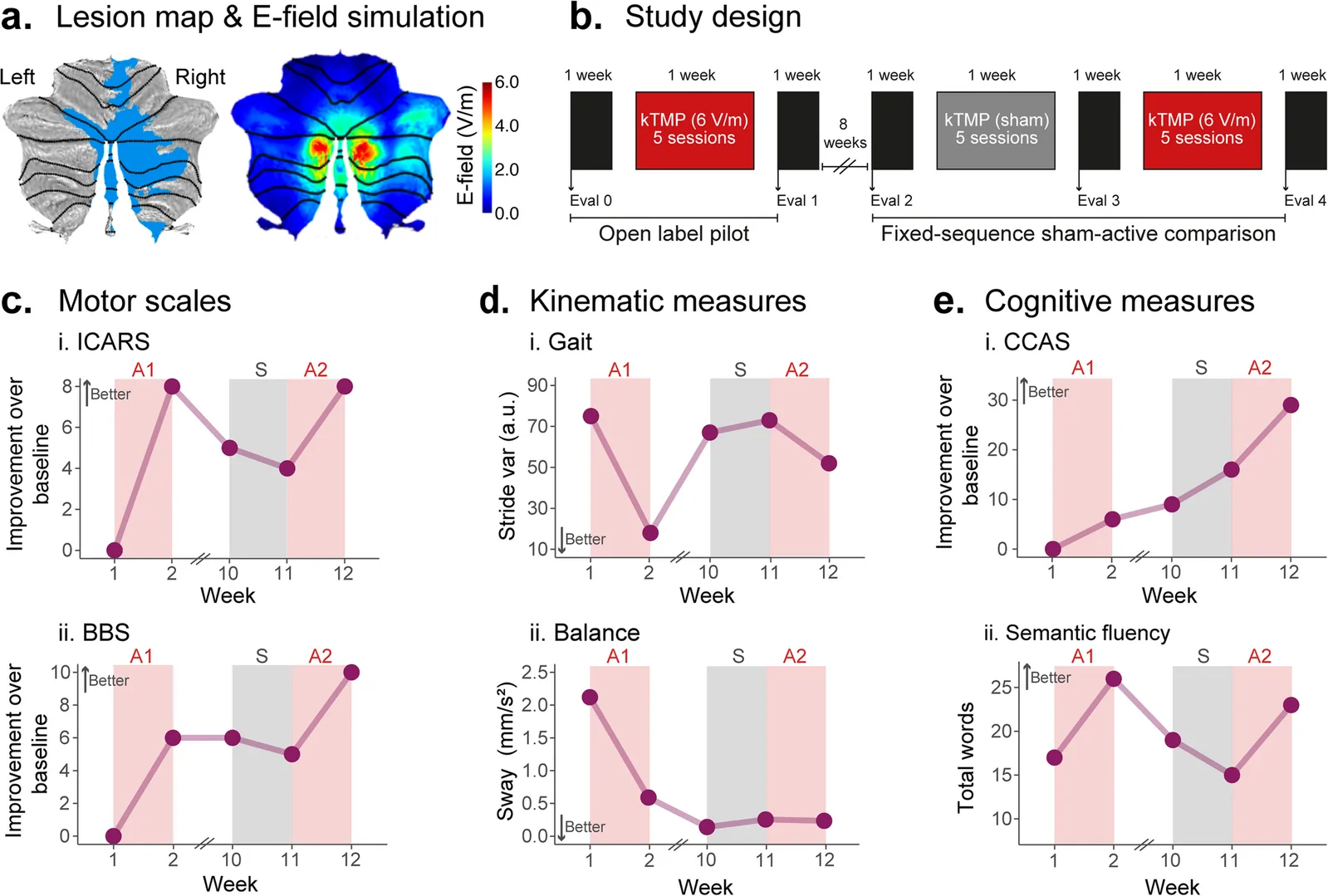
AM-kTMP targeting cerebellar regions produces motor and cognitive improvements in a patient with post-hemorrhagic ataxia.** (A)** Left: Lesion reconstructed from structural MRI and displayed on a cerebellar flat map. Right: Simulated kTMP-induced E-field magnitude and spatial distribution based on template head model. Given the coil position, current was set to induce estimated maximum E-field of ~ 6 V/m, with distribution across the cerebellar vermis, paravermal regions, and right Crus I/II. **(B)** Study design. Phase 1 consisted of a 1-week open-label pilot. After an eight-week break, Phase 2 comprised a fixed-sequence sham-active design with one week of sham stimulation followed by one week of active AM-kTMP. Evaluations (Eval 0–4) were administered at baseline and three days after the final session of each stimulation week. Red shading = active AM-kTMP; gray shading = sham. **(C)** Clinical motor outcomes. ICARS total score (top) and BBS score (bottom) expressed as improvement over baseline (higher = better). A1 = Active Phase 1; S = Sham; A2 = Active Phase 2. **(D)** Kinematic outcomes. Top: Stride time variability, defined as the coefficient of variation of successive stride intervals, where stride onset is identified from heel-strike troughs in the vertical acceleration signal. Bottom: Mediolateral postural sway measured as trunk acceleration along the x-axis during quiet standing with feet together and eyes open. **(E)** Cognitive outcomes. Top: CCAS Scale total score expressed as improvement over baseline (higher = better). Bottom: Total words produced during the semantic fluency component of the CCAS-Scale assessment

The patient is a 78-year-old female with a history of hypertension and hyperlipidemia who sustained a spontaneous focal cerebellar hemorrhage in 2021 (no evidence of vascular malformation or aneurysm). Structural MRI (Fig. 1A) shows a bilateral, midline lesion centered on the cerebellar vermis, extending into the right cerebellar hemisphere and medial aspects of the right deep cerebellar nuclei. At study onset, three years and two months post-hemorrhage, the patient presented with chronic ataxia predominantly affecting postural control and gait, using a walker to help maintain stability when ambulating. Baseline score on the International Cooperative Ataxia Rating Scale (ICARS) was 38/100, with the posture and gait disturbance subscale scoring 18/34, while kinetic function, speech, and oculomotor subscales were 17/52, 3/8, and 0/6, respectively. Complementary assessment with the Berg Balance Scale (BBS) yielded a score of 28/56, consistent with moderate balance impairment and elevated fall risk. Cognitive screening using the Cerebellar Cognitive Affective Syndrome (CCAS) Scale showed a baseline score of 84/120. One task fell below cutoff (category switching), meeting criteria for possible CCAS. Verbal fluency scores were also notably reduced relative to test ceiling. Throughout the study period, the patient’s prescribed medications, comorbid conditions, and use of the walker remained unchanged. She also continued with her regular once-a-week physical therapy sessions during the study period.

### NIBS Parameters

The kTMP system was configured for active stimulation by using a 3.5 kHz carrier frequency signal that was amplitude modulated at 3 Hz, a value chosen based on prior work showing stimulation in this low frequency range promotes inter-regional communication [11]. Based on a template head model, the current from the amplifier was set to produce an E-field of ~ 6 V/m at the most superficial part of the cerebellar cortex (Fig. 1A). This value was previously found to be well-tolerated in a cerebellar-targeted healthy young adult cohort with no difference from sham on sensation ratings [10]. Although the kTMP system can produce stronger E-fields, limiting the intensity to ~ 6 V/m ensured that there was negligible change in coil temperature during the duration of the experiment. The coil was positioned 2 cm below and 1 cm to the right of the inion to target midline regions associated with gait and balance [12] and right Crus I/II, regions associated with language function [13]. Although the E-field overlapped with the lesioned region, it also extended into intact paravermal and Crus I/II tissue. Stimulation was administered in four 5-min blocks for a total of 20 min. During stimulation, the participant performed a verbal fluency task, naming exemplars of a specified category (e.g., work occupations) or phonemic category (e.g., the letter ‘F’) for 1 min, completing approximately 4 categories per 5-minute stimulation block. As coil position could not be maintained during balance exercises, assisted ambulation and supported single-leg stance were performed between blocks.

### Study Design

The case study involved two phases (Fig. 1B). The first phase was an open-label pilot with active AM-kTMP administered on five consecutive days. Three assessments were conducted as part of Phase 1: 17 days prior to the stimulation week (baseline), on the Monday following the stimulation week (three days after the final session), and after an 8-week break with no intervention. The assessments included the ICARS, BBS, and CCAS-S, along with kinematic measures of gait and postural stability obtained with a sternum-mounted inertial measurement unit (IMU) that quantifies spatiotemporal gait and balance parameters from trunk accelerations [14]. To quantify gait, heel-strike and toe-off events were identified according to the pipeline described in [14] and stride time variability was computed as described in [15]. Mediolateral sway during stance (feet together, eyes open, 10-s recording) was quantified as mean acceleration amplitude along the axis of the trunk, averaging leftward and rightward motion. To minimize practice effects, alternate versions of the CCAS-S were used across assessments, with semantic and phonemic fluency categories unique to each timepoint and distinct from those used during stimulation sessions.

One week after the third assessment, the patient returned for Phase 2, a fixed-sequence sham-active phase. This consisted of three weeks, one week of sham stimulation (E-field = 0 V/m), a one-week break, and then a week of active AM-kTMP. Assessments were conducted on the Monday following each stimulation week. Given the case study design, we opted to perform the sham session first to minimize possible saturation effects should the patient exhibit benefits from active stimulation. The same behavioral protocol was maintained throughout, including fluency tasks during stimulation and gait/balance exercises between blocks.

Neither the patient nor experimenters were blinded during Phase 1. Phase 2 was conducted as a double-blind study, including the experimenter who administered and scored the ICARS/BBS scales. Following each active kTMP or sham session, the patient rated sensation, annoyance, pain, and muscle twitches on a 0–10 scale (0 = none, 10 = extreme). Ratings were 0 for all measures across all sessions. The patient’s post-trial report suggested that blinding was preserved during Phase 2.

## Results

### Motor and Kinematic Outcomes

Following the unblinded open-label Phase 1, the patient exhibited marked motor improvement (Fig. 1C, S1, S2). ICARS total score dropped to 30, an 8-point improvement over baseline, with the gains confined to the posture and gait disturbance subscale. Improvements were observed in 5 of 7 items, driven primarily by reductions in body sway and improved gait speed. Kinetic, speech, and oculomotor subscales were unchanged. Similarly, the BBS score improved to 34, a 6-point gain following Phase 1.

Kinematic measures corroborated these clinical gains (Fig. 1D). Stride time variability showed a marked reduction following Phase 1, consistent with a more regular and temporally stable gait pattern. Mediolateral postural sway during standing with feet together and eyes open, measured as trunk acceleration along the x-axis, also decreased following active AM-kTMP.

With the exception of stride variability, the gains were generally maintained over the eight-week interval between Phases 1 and 2. Minimal changes were observed on clinical measures following the sham stimulation week in Phase 2. A second week of active AM-kTMP restored ICARS score to post-Phase 1 levels (30/100) and produced further improvement on the BBS, bringing the total improvement to 10 points above baseline (38/56). Stride time variability similarly improved following active but not sham stimulation. Postural sway, already markedly reduced since Phase 1, remained low throughout Phase 2 with no meaningful change following sham or active stimulation. At the end of the trial, the patient and caregiver also reported anecdotal gains in everyday function (e.g., improved dressing independence and in-home mobility).

### Cognitive Outcomes

The patient also exhibited improvement on cognitive measures over the course of the trial.

(Fig. 1E, S3). CCAS total score improved progressively across all assessment timepoints, increasing by 6 points after Phase 1 (84→90) and reaching a total gain of 29 points by the end of the trial (113/120). By the final assessment, the patient’s diagnostic classification had transitioned from Possible CCAS at baseline to No CCAS. It is important to note that improvement was observed following both active and sham stimulation. As such, practice effects cannot be excluded as one, or perhaps even the sole factor contributing to the overall score trajectory.

While a case study precludes controlling for category- and letter-specific effects, the semantic fluency data from the CCAS point to a more specific benefit from AM-kTMP. Word output increased substantially following Phase 1 (17 to 26 words) and then declined during the eight-week break, with an additional decline in performance following sham stimulation in Phase 2 that brough performance back to baseline level. The patient exhibited a notable improvement (23 words) following the second active stimulation block. Notably, this improvement was not driven by an increase in the initial semantic burst (0–15 s, baseline = 9 words, active Phase 2 = 9 words), but by better word retrieval during the more demanding, later phases of the 1 min epoch (15–60 s, baseline = 8 words, active Phase 2 = 14 words), suggesting that AM-kTMP facilitated sustained semantic retrieval under increasing cognitive demand.

## Discussion

The findings reported here provide initial evidence that AM-kTMP, delivered as part of a combined stimulation and training protocol, can produce clinically meaningful improvements in gait and balance, with kinematic measures providing objective corroboration of the clinical gains. The observed 8-point ICARS improvement in postural control and gait after a single week of stimulation in our case study compares favorably with the mean 5-point improvement reported following 15 consecutive days of rTMS in a sham-controlled trial involving a group of 44 SCA3 patients (4). Although Phase 1 gains were obtained without blinding or sham control, the fixed-sequence sham-active comparison implemented in Phase 2 provides evidence that the observed benefits were specific to when training was combined with AM-kTMP stimulation. Specifically, despite an identical behavioral protocol, performance on the motor measures remained unchanged following sham stimulation but again improved following the subsequent week of active treatment. Furthermore, the likelihood that these effects reflect spontaneous recovery or non-specific trial-related factors is reduced by the chronic nature of the patient’s condition, as all testing was conducted three years post-hemorrhage.

In terms of cognition, the patient showed continuous improvement over the trial on the CCAS scale. We are hesitant to attribute this to the AM-kTMP stimulation given that the substantial improvement observed between the end of Phase 1 stimulation and start of Phase 2 active stimulation; it may well be that the patient benefitted from repeated testing with the CCAS, even though we used different versions of the test at each assessment. The patient’s performance on the semantic fluency component of the CCAS points to a more specific benefit from AM-kTMP. Here the gains were limited to the assessments following active stimulation. Although there is limited flexibility in coil placement when targeting the cerebellum due to its depth, the induced E-field extended into right Crus I/II, regions associated with fluency impairment (13). We note that any claims of specificity regarding fluency changes is post-hoc, just one of a broader set of cognitive measures. As such, this finding should be considered hypothesis-generating rather than confirmatory.

This study has notable limitations, including the single-case design, absence of patient blinding during Phase 1, and fixed sham-before-active sequence in Phase 2. These factors limit control for order effects and claims regarding treatment specificity. Testing was limited to a cerebellar stroke patient, a condition in which the natural clinical course does not involve the progressive decline characteristic of degenerative cerebellar disorders. Mechanistically, we are not positioned to make any inferences about the source of the observed benefits; future studies involving neurophysiological outcome measures would be important to confirm cerebellar circuit engagement.

Nonetheless, the results motivate future testing with larger cohorts, particularly hereditary spinocerebellar ataxias, where the therapeutic need is especially pressing. AM-kTMP can deliver strong, subthreshold frequency-specific stimulation to the cerebellum in a tolerable format, making it a promising candidate for a scalable non-invasive therapeutic approach for a condition that has long lacked effective treatments.

## Supplementary Information

Below is the link to the electronic supplementary material.

Supplementary Material 1

## Data Availability

The data that support the findings of this study are presented within the article and its supplementary materials. Additional data are not publicly available due to patient privacy considerations but are available from the corresponding author upon reasonable request.

## Declarations

### Clinical trial number

Not applicable. This report describes a single-subject case study and does not constitute a prospectively designed interventional trial.
