# Supplementary material for: Motor Improvements Following Non-Invasive Brain Stimulation with kTMP: A Case Study of Cerebellar Stroke
Source: Cerebellum. 2026 Sep 11;25(5):139. doi: 10.1007/s12311-026-02078-z (PMC13569510; doi:10.1007/s12311-026-02078-z)
Supplement: Supplementary file 1 [file 12311_2026_2078_MOESM1_ESM.docx]

**Supplementary Table S1.** International Cooperative Ataxia Rating Scale (ICARS) item and subscale scores across assessment timepoints. Higher scores indicate greater impairment.

| **Task** | **Baseline** | **Post Phase 1 (Active)** | **Follow-up (8 weeks)** | **Post Sham** | **Post Phase 2 (Active)** | **Max Score** |
| --- | --- | --- | --- | --- | --- | --- |
| **I. Posture and gait disturbance** | | | | | | |
| Walking capacities | 4 | 3 | 4 | 4 | 3 | 8 |
| Gait speed | 4 | 2 | 4 | 4 | 2 | 4 |
| Standing capacities (eyes open) | 3 | 2 | 2 | 2 | 2 | 6 |
| Spread of feet in natural position (eyes open) | 1 | 1 | 1 | 1 | 1 | 4 |
| Body sway with feet together (eyes open) | 4 | 1 | 1 | 1 | 1 | 4 |
| Body sway with feet together (eyes closed) | 2 | 1 | 1 | 2 | 1 | 4 |
| Quality of sitting position | 0 | 0 | 0 | 0 | 0 | 4 |
| **Posture and gait score (static score)** | **18** | **10** | **13** | **14** | **10** | **34** |
| **II. Kinetic functions** | | | | | | |
| Right: Knee-tibia test (decomposition of movement) | 2 | 2 | 2 | 2 | 2 | 4 |
| Left: Knee-tibia test (decomposition of movement) | 1 | 1 | 1 | 1 | 1 | 4 |
| Right: Action tremor in the heel-to-knee test | 2 | 2 | 2 | 2 | 2 | 4 |
| Left: Action tremor in the heel-to-knee test | 0 | 0 | 0 | 0 | 0 | 4 |
| Right: Finger-to-nose test (decomposition & dysmetria) | 2 | 2 | 2 | 2 | 2 | 4 |
| Left: Finger-to-nose test (decomposition & dysmetria) | 0 | 0 | 0 | 0 | 0 | 4 |
| Right: Finger-to-nose test (intention tremor of the finger) | 2 | 2 | 2 | 2 | 2 | 4 |
| Left: Finger-to-nose test (intention tremor of the finger) | 0 | 0 | 0 | 0 | 0 | 4 |
| Right: Finger-finger test (action tremor and/or instability) | 2 | 2 | 2 | 2 | 2 | 4 |
| Left: Finger-finger test (action tremor and/or instability) | 1 | 1 | 1 | 1 | 1 | 4 |
| Right: Pronation-supination (alternating movements) | 3 | 3 | 3 | 3 | 3 | 4 |
| Left: Pronation-supination (alternating movements) | 1 | 1 | 1 | 1 | 1 | 4 |
| Drawing (Archimedes spiral) | 1 | 1 | 1 | 1 | 1 | 4 |
| **Kinetic score (limb coordination)** | **17** | **17** | **17** | **17** | **17** | **52** |
| **III. Speech disorders** | | | | | | |
| Dysarthria (fluency of speech) | 1 | 1 | 1 | 1 | 1 | 4 |
| Dysarthria (clarity of speech) | 2 | 2 | 2 | 2 | 2 | 4 |
| **Dysarthria score** | **3** | **3** | **3** | **3** | **3** | **8** |
| **IV. Oculomotor disorders** | | | | | | |
| Gaze-evoked nystagmus | 0 | 0 | 0 | 0 | 0 | 3 |
| Abnormalities of ocular pursuit | 0 | 0 | 0 | 0 | 0 | 2 |
| Dysmetria of the saccade | 0 | 0 | 0 | 0 | 0 | 1 |
| **Oculomotor movement score** | **0** | **0** | **0** | **0** | **0** | **6** |
| **Total ICARS score** | **38** | **30** | **33** | **34** | **30** | **100** |

**Supplementary Table S2.** Berg Balance Scale (BBS) item scores across assessment timepoints. Higher scores indicate better balance performance.

| **Task** | **Baseline** | **Post Phase 1 (Active)** | **Follow-up (8 weeks)** | **Post Sham** | **Post Phase 2 (Active)** | **Max Score** |
| --- | --- | --- | --- | --- | --- | --- |
| Sitting to standing | 4 | 4 | 4 | 4 | 4 | 4 |
| Standing unsupported | 4 | 4 | 4 | 4 | 4 | 4 |
| Sitting with back unsupported but feet supported | 4 | 4 | 4 | 4 | 4 | 4 |
| Standing to sitting | 4 | 4 | 4 | 4 | 4 | 4 |
| Transfers | 4 | 4 | 4 | 4 | 4 | 4 |
| Standing unsupported with eyes closed | 4 | 4 | 4 | 4 | 4 | 4 |
| Standing unsupported with feet together | 0 | 1 | 1 | 1 | 1 | 4 |
| Reaching forward with outstretched arm while standing | 2 | 3 | 3 | 3 | 3 | 4 |
| Pick up object from the floor from a standing position | 0 | 3 | 3 | 3 | 3 | 4 |
| Turning to look behind over left and right shoulders while standing | 2 | 2 | 2 | 2 | 2 | 4 |
| Turn 360 degrees | 0 | 0 | 0 | 0 | 1 | 4 |
| Place alternate foot on step or stool while standing unsupported | 0 | 0 | 0 | 0 | 0 | 4 |
| Standing unsupported one foot in front | 0 | 1 | 1 | 0 | 3 | 4 |
| Standing on one leg | 0 | 0 | 0 | 0 | 1 | 4 |
| **Total score** | **28** | **34** | **34** | **33** | **38** | **56** |

**Supplementary Table S3.** Cerebellar Cognitive Affective Syndrome Scale (CCAS-Scale) subtest scores, number of failed subtests, and diagnostic classification across assessment timepoints. Higher scores indicate better performance.

| **Task** | **Baseline** | | **Post Phase 1 (Active)** | | **Follow-up (8 weeks)** | | **Post Sham** | | **Post Phase 2 (Active)** | | **Max Score** |
| --- | --- | --- | --- | --- | --- | --- | --- | --- | --- | --- | --- |
|  | **Score** | **Fails** | **Score** | **Fails** | **Score** | **Fails** | **Score** | **Fails** | **Score** | **Fails** |  |
| Semantic fluency | 17 | 0 | 26 | 0 | 19 | 0 | 15 | 1 | 23 | 0 | 26 |
| Phonemic fluency | 10 | 0 | 9 | 1 | 11 | 0 | 15 | 0 | 18 | 0 | 19 |
| Category switching | 3 | 1 | 2 | 1 | 6 | 1 | 13 | 0 | 15 | 0 | 15 |
| Digit span forward | 6 | 0 | 7 | 0 | 8 | 0 | 7 | 0 | 7 | 0 | 8 |
| Digit span backward | 6 | 0 | 4 | 0 | 3 | 1 | 5 | 0 | 4 | 0 | 6 |
| Cube | 12 | 0 | 12 | 0 | 15 | 0 | 15 | 0 | 15 | 0 | 15 |
| Verbal recall | 15 | 0 | 14 | 0 | 15 | 0 | 15 | 0 | 15 | 0 | 15 |
| Similarities | 7 | 0 | 8 | 0 | 8 | 0 | 8 | 0 | 8 | 0 | 8 |
| Go no-go | 2 | 0 | 2 | 0 | 2 | 0 | 1 | 0 | 2 | 0 | 2 |
| Affect | 6 | 0 | 6 | 0 | 6 | 0 | 6 | 0 | 6 | 0 | 6 |
| **Total score** | **84** |  | **90** |  | **93** |  | **100** |  | **113** |  | **120** |
| **Total fails** |  | **1** |  | **2** |  | **2** |  | **1** |  | **0** | **10** |
| **Diagnostic label** | **Possible CCAS** | | **Probable CCAS** | | **Probable CCAS** | | **Possible CCAS** | | **No CCAS** | |  |
